# Genomic Feature Analysis of Betacoronavirus Provides Insights Into SARS and COVID-19 Pandemics

**DOI:** 10.3389/fmicb.2021.614494

**Published:** 2021-03-17

**Authors:** Xin Li, Jia Chang, Shunmei Chen, Liangge Wang, Tung On Yau, Qiang Zhao, Zhangyong Hong, Jishou Ruan, Guangyou Duan, Shan Gao

**Affiliations:** ^1^College of Life Sciences, Nankai University, Tianjin, China; ^2^Yunnan Key Laboratory of Stem Cell and Regenerative Medicine, Biomedical Engineering Research Center, Kunming Medical University, Kunming, China; ^3^Taikang Xianlin Drum Tower Hospital, Nanjing University School of Medicine, Nanjing, China; ^4^John Van Geest Cancer Research Centre, School of Science and Technology, Nottingham Trent University, Nottingham, United Kingdom; ^5^School of Mathematical Sciences, Nankai University, Tianjin, China; ^6^School of Life Sciences, Qilu Normal University, Jinan, China

**Keywords:** SARS-CoV-2, MERS-CoV, furin cleavage site, ORF8, recombination

## Abstract

In December 2019, the world awoke to a new betacoronavirus strain named severe acute respiratory syndrome coronavirus-2 (SARS-CoV-2). Betacoronavirus consists of A, B, C and D subgroups. Both SARS-CoV and SARS-CoV-2 belong to betacoronavirus subgroup B. In the present study, we divided betacoronavirus subgroup B into the SARS1 and SARS2 classes by six key insertions and deletions (InDels) in betacoronavirus genomes, and identified a recently detected betacoronavirus strains RmYN02 as a recombinant strain across the SARS1 and SARS2 classes, which has potential to generate a new strain with similar risk as SARS-CoV and SARS-CoV-2. By analyzing genomic features of betacoronavirus, we concluded: (1) the jumping transcription and recombination of CoVs share the same molecular mechanism, which inevitably causes CoV outbreaks; (2) recombination, receptor binding abilities, junction furin cleavage sites (FCSs), first hairpins and *ORF8*s are main factors contributing to extraordinary transmission, virulence and host adaptability of betacoronavirus; and (3) the strong recombination ability of CoVs integrated other main factors to generate multiple recombinant strains, two of which evolved into SARS-CoV and SARS-CoV-2, resulting in the SARS and COVID-19 pandemics. As the most important genomic features of SARS-CoV and SARS-CoV-2, an enhanced *ORF8* and a novel junction FCS, respectively, are indispensable clues for future studies of their origin and evolution. The WIV1 strain without the enhanced *ORF8* and the RaTG13 strain without the junction FCS “RRA**R**” may contribute to, but are not the immediate ancestors of SARS-CoV and SARS-CoV-2, respectively.

## Introduction

A new betacoronavirus strain named severe acute respiratory syndrome coronavirus-2 (SARS-CoV-2) emerged in December 2019 (Hassan et al., 2020a,b; Lundstrom et al., 2020; Seyran et al., 2020). Betacoronavirus consists of A, B, C and D subgroups. Both SARS-CoV and SARS-CoV-2 belong to betacoronavirus subgroup B. Since SARS-CoV-2 is highly similar to SARS-CoV, many studies have focused on the investigation of the receptor binding domain (RBD) of the Spike (S) protein and its receptor angiotensin-converting enzyme 2 (ACE2) using the same strategies and methods as in SARS-CoV (Graham and Baric, 2010). Different from these studies, we previously reported several other findings on SARS-CoV-2 for the first time, including the following in particular: (1) the alternative translation of Nankai coding sequence (Nankai CDS) that characterize the rapid mutation rate of betacoronavirus at the nucleotide level (Chen et al., 2020); (2) a furin cleavage site (FCS) “RRA**R**” in the junction region between S1 and S2 subunits (junction FCS) of SARS-CoV-2 that may increase the efficiency of viral entry into cells (Li et al., 2020); and (3) the use of 5′ untranslated-region (UTR) barcoding for the detection, identification, classification and phylogenetic analysis of—though not limited to—CoVs (Duan et al., 2020). We defined 13–15 nt sequences of 5′ UTRs including the start codons (ATGs) of the first open reading frames (ORFs) as barcodes to represent betacoronaviruses. Using 5′ UTR barcodes, 1,265 betacoronaviruses were clustered into four classes, matching the C, B, A, and D subgroups of betacoronavirus, respectively (Duan et al., 2020). Preliminary experiments showed that the first hairpins (immediately upstream of the first gene *ORF1a*) formed by 5′ UTR barcodes regulate the translation of downstream genes (Li et al., 2021). These previous studies indicated that recombination, receptor binding abilities, junction FCSs and first hairpins are main factors contributing to extraordinary transmission, virulence and host adaptability of betacoronavirus. Particularly, the jumping transcription and recombination of CoVs share the same molecular mechanism (Li et al., 2021), which inevitably causes CoV outbreaks.

In the present study, we started with the identification of key recombination regions and mutation sites in the genomes of betacoronavirus subgroup B and divided the subgroup B into the SARS1 and SARS2 classes using InDels at six sites. Next, we identified two recently detected betacoronavirus strains RmYN01 and RmYN02 from a bat (Zhou et al., 2020) and discovered that RmYN02 was a recombinant SARS2-like CoV strain. This led us to report—for the first time—a recombination event in open reading frame 8 (*ORF8*) at the whole-gene level in a bat, which had been co-infected by two betacoronavirus strains. *ORF8* (Table 1), existing only in betacoronavirus subgroup B, was considered to have played a significant role in adaptation to human hosts following interspecies transmission (Lau et al., 2015) via the modification of viral replication (Muth et al., 2018). Thus, *ORF8* is another main factor contributing to extraordinary transmission, virulence and host adaptability of betacoronavirus. Using the relative RNA abundance between RmYN02 and RmYN01, we validated that *ORF8* associates with viral replication. Finally, we analyzed these genomic features of betacoronavirus in the context of its evolution (conjoint analysis of phylogeny and molecular functions; Liu et al., 2018) to explain the SARS and COVID-19 pandemics.

**TABLE 1:** Annotations of recombination regions and mutation sites.

CDS	Start	End	Length (nt)	Start	End	Length (nt)
ORF1a	266	13,483	13,217	266	13,477	13,212
ORF1b	13,483	21,555	8,073	13,477	21,549	8,073
S	21,563	25,384	3,822	21,556	25,239	3,684
ORF3a	25,393	26,220	828	25,248	26,075	828
E	26,245	26,472	228	26,100	26,327	228
M	26,523	27,191	669	26,378	27,043	666
ORF6	27,202	27,387	186	27,054	27,239	186
ORF7a	27,394	27,759	366	27,246	27,611	366
ORF7b	27,756	27,887	132	27,608	27,739	132
ORF8	27,894	28,259	366	27,746	28,114	369
N	28,274	29,533	1,260	28,116	29,375	1,260
ORF10	29,558	29,674	117	29,400	29,516	117
RC1	3,212	3,337	126	DSQQTVGQQDGSEDNQTTTIQTIVEVQPQLEMELTPVVQTIE		
RC2	3,899	3,955	57	KPFITESKPSVEQRKQDDK		
RC3	21,761	21,796	36	AIHVSGTNGTKR		
RC4	21,971	22,054	84	NDPFLGVYYHKNNKSWMESEFRVYSSAN		
RC5	22,277	22,348	72	QTLLALHRSYLTPGDSSSGWTAGA		
RC6	22,874	22,918	45	SNNLDSKVGGNYNYL		
RC7	22,964	23,020	57	ISTEIYQAGSTPCNGVEGF		
M1	26,109	26,119	11	2,5964	2,5974	11
M2	26,449	−3GAA		26,303	−3GAA	
M3	27,679	−3GAG		27,530	−3GAG	
M4	27,882	−3AAA		27,733	−3AAA	
M5	27,906	−3ATT		27,757	^#^ATT	
M6	29,512	−6AGCTTC		29,353	−6AGCTTC	

**Recombination regions (RC1–7) and mutation sites (M1–6) were annotated in the viral genomes of SARS-CoV-2 (GenBank: MN908947) by column 2–4 and RmYN02 (GISAID: EPI_ISL_412977) by column 5–7. The amino acid sequences are encoded by RC1–7 from SARS-CoV-2. All the insertions and deletions refer to SARS-CoV (GenBank: AY278489). ^#^Since RmYN02 has a recombinant ORF8, it has the same allele at the M5 site as SARS-CoV.**

## Results and Discussion

### Identification of Key Recombination Regions and Mutation Sites

Based on analysis of betacoronavirus subgroup B (section “Materials and Methods”), key insertions and deletions (InDels) were identified at six sites (named M1 to M6) in the *ORF3a*, membrane (*M*), *ORF7a*, *7b*, *8* and nucleocapsid (*N*) genes, respectively (Table 1). Using the InDels at six sites, betacoronavirus subgroup B was divided into two classes: (1) the SARS1 class includes SARS-CoV (from patients) and SARS-like CoV (from animals), and (2) the SARS2 class includes SARS-CoV-2 (from patients) and SARS2-like CoV (from animals). This classification result is simple and reliable as all recombination and mutations between them are unlikely to undergo reversible changes together. As a mutation site, M1 has a length of 8 nt in the SARS1 class and 11 nt in the SARS2 class. M2, M3, M4, and M5 in the SARS2 class have 3-nt deletions that are complete codons, whereas M6 in the SARS2 class has 6-nt deletions that are not complete codons.

Almost all the identified recombination events (Table 1) occurred in the *ORF1a*, *S* and *ORF8* genes. The recombination regions RC1–2 and RC3–7 are located in *ORF1a* and the *S1* region of the *S* gene, respectively, while the recombination events in *ORF8* are complex (see below). To initiate the CoV infection, the S protein encoded by the *S* gene needs to be cleaved into the S1 and S2 subunits for receptor binding and membrane fusion. By analysis of all recombination events in 292 betacoronaviruses of the subgroup B, we obtained the following results: (1) there are a few genotypes of each recombination region (RC1–7); (2) RC3–7 have more diversity than RC1–2 in the genotypes; (3) betacoronaviruses within the SARS1 and SARS2 classes (see above) have the same genotypes of each recombination region; and (4) there are a few non-synonymous substitutions between different sequences of each genotype. These results suggested that recombination, rather than accumulated mutations (i.e., single nucleotide polymorphisms or InDels) had triggered cross-species transmission and outbreaks of SARS-CoV and SARS-CoV-2. Mutations may change potential recombination sites, affecting recombination.

**FIGURE 1:**
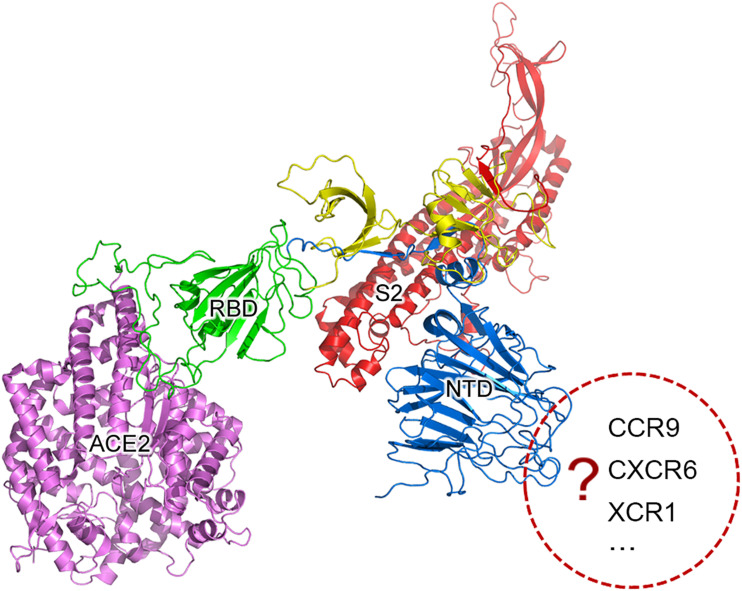
SARS-CoV-2 may have more than one specific receptor. The S protein is cleaved into two subunit S1 and S2 (in red color) for receptor binding and membrane fusion. S1 has two domains, RBD (in green color) and NTD (in blue color). It is well accepted that S1 binds to its specific receptor angiotensin-converting enzyme 2 (ACE2) by the interaction between RBD and ACE2 (in purple color). In the present study, we propose that the S protein of SARS-CoV-2 may have more than one specific receptor for its function like gp120 of HIV has CD4 and CCR5. The structure of S was predicted using trRosetta (Yang et al., 2020).

Further analysis showed that two recombination regions (RC6 and RC7) are localized in the receptor binding domain (RBD) of S1 (Figure 1), while three other recombination regions (RC3, RC4, and RC5) are localized in the N-terminal domain (NTD) of S1. Almost all secondary structures of five protein segments encoded by RC3 to RC7 are disordered, which are responsible for protein protein interaction (PPI). This suggested that the recombination of RC3 to RC7 improve the adaptability of betacoronaviruses in new hosts (host range expansion; Graham and Baric, 2010) by enhancing interaction of RBD and NTD with their receptors. The adaptability improvement may be driven by nature selection, as the positive or negative selection of the *S* gene is particularly strong (Lau et al., 2015). Since both RBD and NTD had similar recombination events in their PPI regions, we proposed that NTD has a specific receptor just like RBD has ACE2. Thus, the S1 subunit of SARS-CoV-2 may have more than one specific receptor (Figure 1) like gp120 of HIV has the receptors of differentiation 4 receptor (CD4) and the C-C chemokine receptor 5 (CCR5). Comprehensive analysis and reuse of data from different sources are necessary to identify the other receptor/s of SARS-CoV-2. A previous study identified two genetic susceptibility loci (rs11385942 at locus 3p21.31 and rs657152 at locus 9q34.2) in COVID-19 patients with respiratory failure using genome-wide association analysis (Ellinghaus et al., 2020). The locus 3p21.31 was associated with six genes *SLC6A20*, *LZTFL1*, *CCR9*, *FYCO1*, *CXCR6*, and *XCR1*. However, the previous study only focused on the further analysis of the locus 9q34.2 to confirm a potential involvement of the ABO blood-group system. The researchers did not notice that three chemokine receptors *CCR9*, *CXCR6*, and *XCR1* merit further investigation as candidates for SARS-CoV-2 receptors. The analysis of bulk RNA-seq data showed high expression of *CCR9* and *XCR1* in thymus and *CXCR6* in T cells, compared to other tissues and cell types (Ellinghaus et al., 2020). In particular, the thymic cells were consistently negative for ACE2 but many CoVs can infect thymus (Lins and Smaniotto, 2020). By investigating interaction of three protein segments encoded by RC3 to RC5 in NTD (Table 1) with *CCR9*, *CXCR6*, and *XCR1*, we found that *CCR9* is the most possible candidate among three chemokine receptors. However, the final determination of the other receptor/s of SARS-CoV-2 needs more calculation and experiments on candidates at the whole-genome level. Our study did not rule out the possibilities of non-receptor proteins binding to NTD.

### Identification of Two Betacoronavirus Strains From a Bat

Recently, two betacoronavirus strains RmYN01 and RmYN02 (GISAID: EPI_ISL_412976 and EPI_ISL_412977) were detected from a bat of *Rhinolophus malayanus* (Zhou et al., 2020). Since betacoronaviruses of the subgroup B share many highly similar regions in their genome sequences, it is difficult to assemble them correctly from a mixed sample using short high-throughput sequencing (HTS) reads. Therefore, EPI_ISL_412976 was only assembled into a partial sequence in that previous study (Zhou et al., 2020). However, the exact identification of viruses requires the complete genomes or even the full-length genomes. Using paired-end sequencing data, we reassembled these two virus genomes and obtained two full-length sequences to update EPI_ISL_412976 and EPI_ISL_412977 (Supplementary Material). Using 5′ UTR barcodes (section “Introduction”), the betacoronaviruses RmYN01 and RmYN02 were identified as belonging to the subgroup B. Using the InDels at M1–M6, RmYN01 was further identified as belonging to the SARS1 class, respectively. Using the InDels at M1–M4 and M6, RmYN02 was further identified as belonging to the SARS2 class but a recombinant SARS2-like CoV strain. RmYN02 was supposed to have a 3-nt deletion at the M5 site; however, it did not (Table 1). This led us to report—for the first time—a recombination event in *ORF8* at the whole-gene level in a bat, which had been co-infected by two betacoronavirus strains.

Existing only in betacoronavirus subgroup B, *ORF8* was considered to associate with viral replication (section “Introduction”), mainly based on the discovery of a 29-nt deletion in SARS-CoV (GenBank: AY274119) (Muth et al., 2018) and a 382-nt deletion in SARS-CoV-2 (GISAID: EPI_ISL_414378-80) (Su et al., 2020). However, it was also reported that *ORF8* associated with attenuation without changes in its replication (Young et al., 2020). Although many recombination events in *ORF8* of betacoronaviruses have been reported by sequence analysis, it is difficult to determine whether they were recombination events or small-size mutation (InDel and SNP) accumulations as most of them only occurred over very small genomic regions, except the two events (see above). In the present study, the discovery of a recombination event in *ORF8* at the whole-gene level led to the determination of three types (see below) of *ORF8* genes in betacoronavirus subgroup B, providing new clues to investigate the functions of *ORF8*.

**FIGURE 2:**
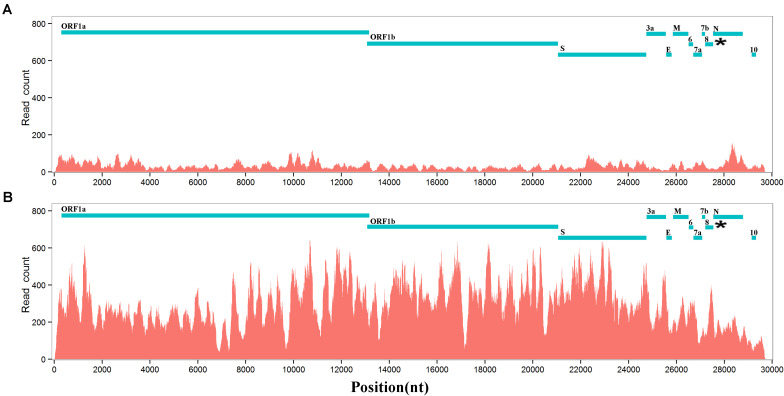
RNA abundances of RmYN01 and RmYN02 in a bat. RNA-seq data from a bat was aligned to two genomes of RmYN01 and RmYN02 (GISAID: EPI_ISL_412976 and EPI_ISL_412977). RNA abundance is represented by read counts (*y*-axis). The relative RNA abundance between RmYN02 and RmYN01 was about 9. **(A)** RmYN01 was identified as belonging to the SARS1 class and has a type 3 *ORF8*. **(B)** RmYN02 was identified as belonging to the SARS2 class but has an type 2 *ORF8* (enhanced *ORF8*).

Next, we conducted further research on the biological functions of *ORF8* to test a previous hypothesis that type 2 *ORF8* genes enhance the viral replication. RmYN01 and RmYN02 were simultaneously detected in a bat, providing a special opportunity to compare their genome copy numbers. The difference between the genome copy numbers of RmYN01 and RmYN02 can be estimated by their relative RNA abundance. Aligning RNA-seq data to the genomes of RmYN01 and RmYN02, our calculation showed that the RmYN01 genome was covered 99.85% of its length with an average depth of 32.89 (Figure 2A), while the RmYN02 genome was covered 99.89% with an average depth of 298.99 (Figure 2B). The relative RNA abundance between RmYN02 and RmYN01 was about 9. Based on the “leader-to-body fusion” model explaining the replication and transcription of CoVs (Li et al., 2021), the difference of RNA abundance within the *ORF1a* and *ORF1b* regions (Figures 2A,B) resulted from CoV replication, rather than transcription. This result suggests that type 2 *ORF8* (named enhanced *ORF8*) genes enhance the replication of RmYN02, ruling out the possibility that transcription contributes to the relative RNA abundance between RmYN02 and RmYN01. Another factor *S1* may also result in the difference of genome copy numbers between of RmYN01 and RmYN02. However, the nucleotide identity of *S1* gene regions between RmYN01 and RmYN02 reaches 69%, while type 3 *ORF8* of RmYN01 and type 2 *ORF8* of RmYN02 cannot be well aligned to calculate the nucleotide identity (see below). The difference in *S1* is nothing compared with that in *ORF8*. These results validated that *ORF8* associates with viral replication.

### Conjoint Analysis of Phylogeny and Molecular Functions

**FIGURE 3:**
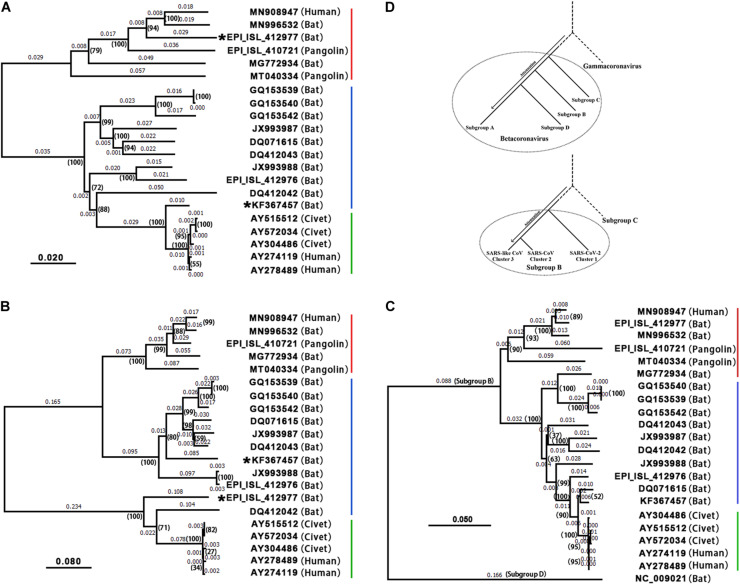
Phylogenetic analysis and evolution of betacoronavirus. The accession numbers of the GenBank or GISAID databases were used to represent the viral genomes: MN908947: SARS-CoV-2; MN996532: the SARS2-like CoV strain RaTG13; EPI_ISL_412977: the SARS2-like CoV strain RmYN02; EPI_ISL_412976: the SARS-like CoV strain RmYN01; KF367457: the SARS-like CoV strain WIV1; AY274119: the SARS-CoV strain Tor2; AY278489: the SARS-CoV strain GD01. Decimal above the branches are phylogenetic distances calculated using the NJ method with a bootstrap test (1,000 replicates). The bootstrap values (indicated by parentheses) were in the format for displaying percentages with “%” omitted. 19 of 21 betacoronaviruses were classified into the SARS-CoV-2&SARS2-like CoV (red), SARS-like CoV (blue) and SARS-CoV (green) clusters, while the other two (i.e., WIV1 and RmYN02) are recombinant strains. **(A)** Phylogenetic tree 1 was built using large segments spanning *S2*, *ORF3a*, *E*, *M*, *ORF6*, *7a*, *7b*, *N(9b)*, and *ORF10* (Table 1). **(B)** Phylogenetic tree 2 was built using *ORF8*. As type 2 *ORF8* genes cannot be well aligned to types 1 or 3 *ORF8* genes to calculate nucleotide identities, the distances between the SARS-CoV cluster and the SARS-CoV-2&SARS2-like CoV or SARS-like CoV clusters are not accurate. **(C)** Phylogenetic tree 3 was built using CDSs of *nsp12* (RNA-dependent RNA polymerase, RdRP). HKU9-CoV (RefSeq: NC_009021) from the subgroup D was used as an outgroup strain. **(D)** MERS-CoV (GenBank: JX869059), SARS-CoV-2 (GenBank: MN908947), HKU9-CoV (RefSeq: NC_009021), MHV (RefSeq: NC_001846) and IBV (RefSeq: NC_001451) were used to represent betacoronavirus subgroups C, B, D, A and gammacoronavirus, respectively in the upper phylogenetic tree; SARS-CoV-2 (GenBank: MN908947), SARS-CoV (GenBank: AY278489), RmYN01 (GISAID: EPI_ISL_412976), and MERS-CoV (GenBank: JX869059) were used to represent the SARS-CoV-2&SARS2-like CoV, SARS-CoV, and SARS-like CoV clusters and the betacoronavirus subgroup C, respectively, in the lower phylogenetic tree.

Based on conjoint analysis of phylogeny and molecular functions that was proposed in our previous study (Liu et al., 2018), genes (i.e., *ORF1a*, *S1*, and *ORF8*) containing the recombination regions under high selection pressure must be removed in phylogenetic analysis. Using large segments (Supplementary Material) spanning *S2*, *ORF3a*, envelope (*E*), *M*, *ORF6*, *7a*, *7b*, *N(9a)*, and *ORF10* (Table 1), phylogenetic tree 1 (Figure 3A) showed that 19 of 21 betacoronaviruses (section “Materials and Methods”) were classified into two major clades, corresponding to the SARS1 and SARS2 classes (see above) divided using the InDels at six sites, respectively: (1) the SARS1 class includes two clusters—the SARS-CoV cluster including SARS-CoV and a few most closely related SARS-like CoVs (from bats or civets) and the SARS-like CoV cluster including all other SARS-like CoVs; and (2) the SARS2 class includes two clusters—the SARS-CoV-2 cluster and the SARS2-like cluster including all SARS2-like CoVs (from bats and pangolins). The SARS1 class was divided into the SARS-CoV and SARS-like CoV clusters by the types of ORF8, while the SARS2 class was divided into the SARS-CoV-2 and SARS2-like CoV clusters by the presence of junction FCS “RRA**R**” (Li et al., 2020). Currently, the SARS-CoV-2 cluster only includes one strain (GenBank: MN908947), which was clustered with SARS2-like CoV into one clade in phylogenetic trees.

Comparing phylogenetic tree 1 (Figure 3A) using large segments with 2 (Figure 3B) using only *ORF8* genes (Supplementary Material), all betacoronaviruses were consistently classified into the same clusters in both trees, except RmYN02 and the SARS-like CoV strain WIV1 (GenBank: KF367457). However, tree 2 did not reflect the evolutionary relationship of 21 strains due to the recombination events of *ORF8*. *ORF8* and other genomic regions of betacoronavirus subgroup B have different origins (Lau et al., 2015). Using 21 CDSs of *nsp12* (RNA-dependent RNA polymerase, RdRP), the rooted phylogenetic tree 3 (Figure 3C) was construct to confirm the evolutionary relationship of betacoronavirus strains in tree 1 (Figure 3A). In phylogenetic tree 2, the SARS-CoV-2&SARS2-like CoV, SARS-CoV and SARS-like CoV clusters have types 1, 2, and 3 ORF8 genes, respectively. Type 1 *ORF8* genes possess low nucleotide identities (below 70%) to type 3 *ORF8* genes, while type 2 *ORF8* genes are so highly divergent from types 1 and 3 *ORF8* genes that they cannot be well aligned to calculate nucleotide identities between them. Since RmYN02 belongs to the SARS2 class (Figure 3A) but has a type 2 rather than type 1 *ORF8* (Figure 3B), RmYN02 was identified as a recombinant SARS2-like CoV strain. The identification of RmYN02 indicated that recombination occurred across the SARS1 and SARS2 classes, which has potential to generate a new strain with similar risk as SARS-CoV and SARS-CoV-2.

As a recombinant SARS-like CoV strain with a type 3 *ORF8* isolated from Chinese horseshoe bats (*Rhinolophus sinicus*), WIV1 was considered most closely related to SARS-CoV (Ge et al., 2013). Comparing phylogenetic tree 1 with 2 suggested that WIV1 is not the immediate ancestor of SARS-CoV. This confirmed a previous hypothesis: the ancestor of SARS-like CoVs from civets was a recombinant virus with *ORF8* originating from greater horseshoe bats (*Rhinolophus ferrumequinum*) and other genomic regions originating from different horseshoe bats (Lau et al., 2015). However, whether these recombination events occurred in bats or civets remains unclear (Lau et al., 2015). Both phylogenetic tree 1 (Figure 3A) and 2 (Figure 3B) consistently revealed that SARS-CoV-2 is most closely related to the well-known strain RaTG13 (GenBank: MN996532) isolated from intermediate horseshoe bats (*Rhinolophus affinis*). However, RaTG13 is unlikely to be the immediate ancestor of SARS-CoV-2 due to lack of the junction FCS “RRA**R**.” In addition, all pangolin (*Manis javanica*) betacoronaviruses investigated using their public genomes (section “Materials and Methods”) were identified as belonging to the SARS2-like CoV cluster. However, further analyses of these genomes did not support that pangolins are the intermediate host(s) of SARS-CoV-2 (Lam et al., 2020) for three main reasons: (1) pangolin betacoronaviruses do not have the junction FCS “RRA**R**” (Li et al., 2020); (2) all reported strains (e.g., GISAID: EPI_ISL_410721 and GenBank: MT040334) are farther from SARS-CoV-2 than RaTG13 in the phylogenetic tree 1 and 3; and (3) pangolin betacoronaviruses are unlikely to lose “RRA**R**” so soon, as betacoronaviruses of the subgroup A lost junction FCSs after a long-term evolutionary change. This suggested that the intermediate host(s) of SARS-CoV-2 carry at least one betacoronavirus strain with junction FCS “RRA**R**” in the S protein.

### Outbreak and Evolution of Betacoronavirus

Recombination, receptor binding abilities, junction FCSs, first hairpins and *ORF8*s (see above) are main factors contributing to extraordinary transmission, virulence and host adaptability of betacoronavirus. By analysis of these main factors in 1,300 betacoronavirus genomes (section “Materials and Methods”), we concluded: (1) as the most important factor, rapid recombination of viral genomes provides CoVs the strong ability of cross-species transmission and outbreak; (2) the strong recombination ability of CoVs integrated other main factors to generate multiple recombinant strains, of which very a few evolved into super virus strains (e.g., SARS-CoV and SARS-CoV-2) causing pandemics by natural selection; (3) the immediate ancestor of betacoronavirus did most likely have two junction FCS and a strong first hairpin, and it transmitted across species during its outbreak; and (4) after a period of adaption in new hosts, betacoronavirus was attenuated to spread widely and persist in the host population by loss of abilities attributed to one or more factors (e.g., junction FCSs).

**FIGURE 4:**
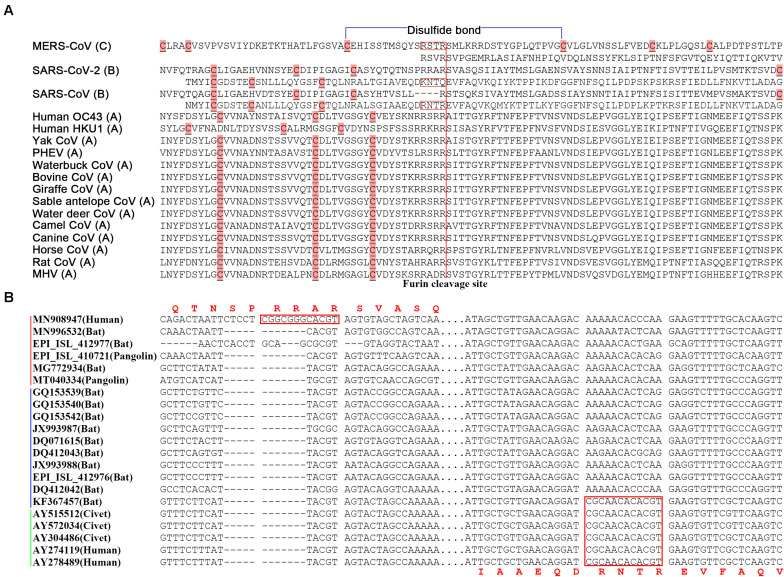
Junction furin cleavage sites of betacoronaviruses. **(A)** Two regions having potential to contain junction furin cleavage sites (FCSs) are showed for MERS-CoV (R694 and R751), SARS-CoV-2 (R685 and Q779) and SARS-CoV (R667 and R761), while only one region is showed for other betacoronaviruses. Junction FCSs (in red box) are non-functioning, lost or inaccessible due to different reasons. The disulfide bond (in blue color) is only across “RSTR” of MERS-CoV. MERS-CoV (GenBank: JX869059) belongs to the subgroup C; SARS-CoV (GenBank: AY278489) and SARS-CoV-2 (GenBank: MN908947) belong to the subgroup B; Human OC43 (GenBank: KF530084), Human HKU1 (GenBank: KF686346), Yak CoV (GenBank: MH810163), PHEV (GenBank: KY419107), Waterbuck CoV (GenBank: FJ425186), Bovine CoV (GenBank: MH043954), Giraffe CoV (GenBank: EF424622), Sable antelope CoV (GenBank: EF424621), Water deer CoV (GenBank: MG518518), Camel CoV (GenBank: MN514963), Canine CoV (GenBank: JX860640), Horse CoV (GenBank: LC061274), Rat CoV (GenBank: JF792617) and MHV (GenBank: AF029248) belong to the subgroup A. PHEV: porcine hemagglutinating encephalomyelitis virus; MHV: mouse hepatitis virus. **(B)** Two regions having potential to contain junction FCSs (between spaces) are showed at the nucleotide level. The SARS-CoV-2&SARS2-like CoV, SARS-like CoV and SARS-CoV clusters were indicated by red, blue and green lines.

In betacoronavirus subgroup C (Figure 4A), middle east respiratory syndrome coronavirus (MERS-CoV) has two junction FCSs. The first one “RST**R,**” located at position 694 in the S protein (noted as MERS-S-R694), is non-functioning, as a result of attenuation, because there is a disulfide bond across MERS-S-R694. However, the second junction FCS “RSV**R**” (MERS-S-R751) is still functional. Originated from the same ancestor of MERS-CoV, MERS-like CoVs (e.g., hedgehog CoV) without “RST**R**” were further attenuated by loss of MERS-S-R751. In betacoronavirus subgroup B (Figures 4A,B), SARS-CoV-2 (GenBank: MN908947) has the junction FCS “RRA**R**” (SARS2-S-R685), but lost another junction FCS by substituting “KNTQ” for “RNT**R**” (SARS-S-R761), as a result of attenuation. All SARS-2 like CoVs (from bats or pangolins; Li et al., 2020) without “RNT**R**” were further attenuated by loss of SARS2-S-R685. The immediate ancestor of SARS-CoV with inaccessible “RNT**R**” (SARS-S-R761) that has secondary structures in helix rather than coil was an attenuated variant of SARS-CoV-2 by loss of “RRA**R**” (SARS2-S-R685). All SARS-like CoVs without “RNT**R**” were further attenuated by loss of “RRA**R**.” In betacoronavirus subgroup D, HKU9-CoV was attenuated by loss of two junction FCSs but still have a strong first hairpin. In betacoronavirus subgroup A (Figure 4A), although almost all strains (e.g., HCoV-OC43 and HCoV-HKU1) still have one junction FCS, they do not have strong first hairpins or enhanced *ORF8*s. These strains were heavily attenuated from the immediate ancestor of betacoronavirus due to complex reasons. For an example, the average arginine (R) percentage (2.63%) of S proteins in betacoronaviruses of the subgroup A except mouse hepatitis virus (MHV) is significantly lower than those in MHV and betacoronaviruses of the subgroups B, C, and D (3.34, 3.33, 3.32, and 3.33%). This indicated that accumulated mutations caused attenuation by loss of arginine residues, since arginine residues are indispensable for the protease cleavage sites. Other reasons may include the loss of strong first hairpins and genetic events in the transcription regulatory sequences (Li et al., 2021), an important factor that was not further investigated in the present study, but merit further investigation in the future.

Guided by conjoint analysis of phylogeny and molecular functions, we concluded the following (Figure 3D): (1) in general, betacoronaviruses (and even CoVs) were and are undergoing attenuation to spread widely and persist in host population after every outbreak; (2) the immediate ancestor of the subgroup C (e.g., MERS-CoV) was most closely related to the immediate ancestor of betacoronavirus with slight attenuation; (3) the immediate ancestors of the subgroups B and D diverged subsequently and were further attenuated; and (4) betacoronaviruses of the subgroup A were most heavily attenuated and have the highest diversity in their genomes and hosts. In betacoronavirus subgroup B (Figure 3D), (1) the immediate ancestor of the SARS-CoV-2 cluster was most closely related to the immediate ancestor of the subgroup B with slight attenuation; (2) the immediate ancestor of the SARS-CoV cluster diverged subsequently and was further attenuated; and (3) the SARS-like CoV cluster was most heavily attenuated and has the highest diversity in the genomes and hosts. All the SARS-like CoVs (e.g., WIV1 and RmYN01) are attenuated variants of SARS-CoV, while all the SARS2-like CoVs (e.g., RaTG13, RmYN02 and betacoronaviruses from pangolins) are attenuated variants of SARS-CoV-2. As recombinant betacoronavirus, the immediate ancestor of SARS-CoV is characterized by the enhanced *ORF8*, while the immediate ancestor of SARS-CoV-2 is characterized by the junction FCS “RRA**R**.” Therefore, WIV1 without the enhanced *ORF8* and RaTG13 without the junction FCS “RRA**R**” may contribute to, but are not the immediate ancestors of SARS-CoV and SARS-CoV-2, respectively.

## Conclusion

Recombination, receptor binding abilities, junction FCSs, first hairpins and *ORF8*s are main factors contributing to extraordinary transmission, virulence and host adaptability of betacoronavirus. Junction FCSs and enhanced *ORF8*s increase the efficiencies in viral entry into cells and genome copy numbers, respectively, while strong first hairpins may enhance the translation of their downstream proteins. The strong recombination ability of CoVs integrated other main factors to generate multiple recombinant strains, two of which evolved into SARS-CoV and SARS-CoV-2 by natural selection, resulting in the SARS and COVID-19 pandemics. The outbreaks of MERS-CoV, SARS-CoV and SARS-CoV-2 were triggered by recombination events, not accumulated mutations. So it is not suitable to estimate their divergence time using current theories in evolutionary biology. The origins of *ORF8* and the junction FCS “RRA**R**” are still unknown. Future investigation needs be conducted to search for the betacoronavirus strains that provided the enhanced *ORF8* and the junction FCS “RRA**R**” to SARS-CoV and SARS-CoV-2, respectively. Based on our theories, two predictions can be made: (1) more attenuated (by loss of junction FCSs or *ORF8*s) variants of SARS-CoV-2 will be reported; and (2) SARS2-like CoV with at least one junction FCS “RRA**R**” will be eventually detected.

## Materials and Methods

The software VirusDetect (Zheng et al., 2017) was used to detect viruses in RNA-seq data (Zhou et al., 2020). The software Fastq_clean (Zhang et al., 2014) was used for RNA-seq data cleaning and quality control. The genomes of RmYN01 and RmYN02 (GISAID: EPI_ISL_412976 and EPI_ISL_412977) were reassembled by aligning RNA-seq data on two closest reference genomes JX993988 and MN908947. SVDetect v0.8b and SVFilter (Zhang et al., 2016) were used to removed abnormal aligned reads. Several haploid contigs (Supplementary Material) highly similar to the complete RmYN01 genome were also assembled. This suggested that there exists more than one betacoronavirus strain belonging to the SARS-like CoV cluster in the same sample, from which RmYN01 and RmYN02 were detected. Protein structure data (PDB: 5 × 5B, 6ZGE, 6ZGF, 5 × 5F, 3JCL, 5I08, and 6OHW) were used to analyzed the FCSs of SARS-COV, SARS-CoV-2, SARS2-like CoV, MERS-CoV, MHV, HCoV-HKU1, and HCoV-HKU43, respectively.

1,265 genome sequences of betacoronaviruses (in subgroups A, B, C, and D) were downloaded from the NCBI Virus database^1^ in our previous study (Li et al., 2020). Among these genomes, 292 belongs to betacoronavirus subgroup B. Plus 35 genomes from the GISAID database, 1,300 betacoronavirus genomes were used for analysis in the present study. In our previous study, 10 complete genomes of betacoronavirus subgroup B (GenBank: JX993987, JX993988, GQ153539, GQ153540, GQ153542, DQ071615, DQ412043, AY515512, AY572034, and DQ497008) were selected and used for the analysis (Liu et al., 2018). To trace the origin of SARS-CoV, five complete genomes were added in the present study. They are DQ412042 (SARS-like CoV from *Rhinolophus ferrumequinum*), AY274119 (SARS-like CoV from a SARS patient in Toronto, Tor2), AY278489 (SARS-like CoV from a SARS patient in Guangdong, GD01), AY304486 (SARS-like CoV from civet) and KF367457 (SARS-like CoV from bat). DQ497008 was removed as a redundant sequence of AY274119 and AY278489. To trace the origin of SARS-CoV-2, three complete genomes were added. They are MN908947 (SARS-CoV-2), MN996532 (SARS2-like CoV hosted in Intermediate Horseshoe bats (*Rhinolophus affinis*) from Yunnan) and MG772934 (SARS2-like CoV hosted in Chinese horseshoe bats (*Rhinolophus sinicus*) from Zhejiang). A SARS2-like CoV (GISAID: EPI_ISL_410721) from pangolins (Collected in Guangdong, China) and a SARS2-like CoV (GenBank: MT040334) from pangolins (Collected in Guangxi, China) were used to represent SARS2-like CoVs from pangolins after the removal of sequence redundancy. In total, 21 complete genomes including RmYN01 and RmYN02 (GISAID: EPI_ISL_412976 and EPI_ISL_412977) were used for the phylogenetic analysis applying the neighbor joining (NJ) method. Sequence alignment was performed using the Bowtie v0.12.7 software with paired-end alignment allowing 3 mismatches; mutation detection and other data processing were carried out using Perl scripts; the phylogenetic analysis was performed using MEGA v7.0.26; Statistics and plotting were conducted using the software R v2.15.3 with the Bioconductor packages (Gao et al., 2014). The structure of the S protein was predicted using trRosetta (Yang et al., 2020).

## Data Availability Statement

The datasets presented in this study can be found in online repositories. The names of the repository/repositories and accession number(s) can be found in the article/Supplementary Material.

## Author Contributions

SG conceived the project. SG and GD supervised this study. JC and SC conducted programming. XL, LW, and QZ downloaded, managed, and processed the data. TY predicted the structure of the S protein. JR analyzed the structure of S1. SG drafted the main manuscript text. SG and ZH revised the manuscript. All authors contributed to the article and approved the submitted version.

## Conflict of Interest

The authors declare that the research was conducted in the absence of any commercial or financial relationships that could be construed as a potential conflict of interest.

## Footnotes

1 https://www.ncbi.nlm.nih.gov/labs/virus

## References

[B1] Chen J. Shi J. Yau T. O. Liu C. Li X. Zhao Q. (2020). Bioinformatics analysis of the 2019 novel coronavirus genome. *Chin. J. Bioinform.* 18 96–102. (In Chinese)

[B2] Duan G. Shi J. Xuan Y. Chen J. Liu C. Ruan J. (2020). 5′ UTR barcode of the 2019 novel coronavirus leads to insights into Its virulence. *Chin. J. Viro.* 36 365–369. (In Chinese)

[B3] Ellinghaus D. Degenhardt F. Bujanda L. Buti M. Albillos A. Invernizzi P. (2020). Genomewide association study of severe Covid-19 with respiratory failure. *N. Engl. J. Med.* 383 1522–1534. 10.1056/NEJMoa2020283 32558485 PMC7315890

[B4] Gao S. Ou J. Xiao K. (2014). *R Language and Bioconductor in Bioinformatics Applications (Chinese Edition).* Tianjin: Tianjin Science and Technology Translation Publishing Ltd.

[B5] Ge X.-Y. Li L.-J. Yang X.-L. Chmura A. A. Zhu G. Epstein J. H. (2013). Isolation and characterization of a bat SARS-like coronavirus that uses the ACE2 receptor. *Nature* 503 535–538. 10.1038/nature12711 24172901 PMC5389864

[B6] Graham R. L. Baric R. S. (2010). Recombination, reservoirs, and the modular spike: mechanisms of Coronavirus cross-species transmission. *J. Virol.* 84 3134–3146. 10.1128/JVI.01394-09 19906932 PMC2838128

[B7] Hassan S. S. Attrish D. Ghosh S. Choudhury P. P. Uversky V. N. Uhal B. D. (2020a). Notable sequence homology of the ORF10 protein introspects the architecture of SARS-COV-2. *bioRxiv* [Preprint]. 10.1101/2020.09.06.284976 PMC8051021 33862077

[B8] Hassan S. S. Ghosh S. Attrish D. Choudhury P. P. Seyran M. Pizzol D. (2020b). A unique view of SARS-CoV-2 through the lens of ORF8 protein. *bioRxiv* [Preprint]. 10.1101/2020.08.25.267328 PMC8049180 33872970

[B9] Lam T. T.-Y. Jia N. Zhang Y.-W. Shum M. H.-H. Jiang J.-F. Zhu H.-C. (2020). Identifying SARS-CoV-2-related coronaviruses in malayan pangolins. *Nature* 583 282–285. 10.1038/s41586-020-2169-0 32218527

[B10] Lau S. K. P. Feng Y. Chen H. Luk H. K. H. Yang W. H. Li K. S. M. (2015). Severe acute respiratory syndrome (SARS) coronavirus ORF8 protein is acquired from SARS-related coronavirus from greater horseshoe bats through recombination. *J. Virol.* 89 10532–10547. 10.1128/JVI.01048-15 26269185 PMC4580176

[B11] Li X. Duan G. Zhang W. Shi J. Chen J. Chen S. (2020). Furin cleavage site was discovered in the S protein of the 2019 novel coronavirus. *Chin. J. Bioinform.* 18 103–108. (In Chinese)

[B12] Li X. Cheng Z. Wang F. Chang J. Zhao Q. Zhou H. (2021). A negative feedback model to explain regulation of SARS-CoV-2 replication and transcription. *Front. Gene.* 12:263327. 10.3389/fgene.2021.641445 PMC7954359 33719350

[B13] Lins M. P. Smaniotto S. (2020). Potential impact of SARS-CoV-2 infection on the thymus. *Can. J. Microbiol.* 67 23–28. 10.1139/cjm-2020-0170 32640169

[B14] Liu C. Chen Z. Hu Y. Ji H. Yu D. Shen W. (2018). Complemented Palindromic small RNAs first discovered from SARS coronavirus. *Genes* 9:442. 10.3390/genes9090442 30189613 PMC6162610

[B15] Lundstrom K. Seyran M. Pizzol D. Adadi P. El-Aziz T. M. A. Hassan S. S. (2020). The importance of research on the origin of SARS-CoV-2. *Viruses* 12:1203. 10.3390/v12111203 33105685 PMC7690418

[B16] Muth D. Corman V. M. Roth H. Binger T. Dijkman R. Gottula L. T. (2018). Attenuation of replication by a 29 nucleotide deletion in SARS-coronavirus acquired during the early stages of human-to-human transmission. *Sci. Rep.* 8:15177. 10.1038/s41598-018-33487-8 30310104 PMC6181990

[B17] Seyran M. Pizzol D. Adadi P. El-Aziz T. M. A. Hassan S. S. Soares A. (2020). Questions concerning the proximal origin of SARS-CoV-2. *J. Med. Virol.* 93 1204–1206. 10.1002/jmv.26478 32880995 PMC7898912

[B18] Su Y. C. Anderson D. E. Young B. E. Zhu F. Linster M. Kalimuddin S. (2020). Discovery of a 382-nt deletion during the early evolution of SARS-CoV-2. *bioRxiv* [Preprint]. 10.1101/2020.03.11.987222 PMC7374062 32694143

[B19] Yang J. Anishchenko I. Park H. Peng Z. Baker D. (2020). Improved protein structure prediction using predicted interresidue orientations. *Proc. Natl. Acad. Sci.U.S.A.* 117 1496–1503. 10.1073/pnas.1914677117 31896580 PMC6983395

[B20] Young B. E. Fong S.-W. Chan Y.-H. Mak T.-M. Ang L. W. Anderson D. E. (2020). Effects of a major deletion in the SARS-CoV-2 genome on the severity of infection and the inflammatory response: an observational cohort study. *Lancet* 396 603–611. 10.1016/S0140-6736(20)31757-8 32822564 PMC7434477

[B21] Zhang F. Xu T. Mao L. Yan S. Chen X. Wu Z. (2016). Genome-wide analysis of dongxiang wild rice (Oryza rufipogon Griff.) to investigate lost/acquired genes during rice domestication. *BMC Plant Biol.* 16:103. 10.1186/s12870-016-0788-2 27118394 PMC4845489

[B22] Zhang M. Zhan F. Sun H. Gong X. Fei Z. Gao S. (2014). “Fastq_clean: an optimized pipeline to clean the Illumina sequencing data with quality control,” in *Proceedings of the 2014 IEEE International Conference on Bioinformatics and Biomedicine (BIBM)*, Belfast, 44–48. 10.1109/BIBM.2014.6999309

[B23] Zheng Y. Gao S. Padmanabhan C. Li R. Galvez M. Gutierrez D. (2017). VirusDetect: an automated pipeline for efficient virus discovery using deep sequencing of small RNAs. *Virology* 500 130–138. 10.1016/j.virol.2016.10.017 27825033

[B24] Zhou H. Chen X. Hu T. Li J. Song H. Liu Y. (2020). A novel bat coronavirus closely related to SARS-CoV-2 contains natural insertions at the S1/S2 cleavage site of the spike protein. *Curr. Biol.* 30 2196–2203. 10.1016/j.cub.2020.05.023 32416074 PMC7211627

